# Association Between a Flat Oral Glucose Tolerance Test Pattern and Small-for-Gestational-Age Infants: A Retrospective Cohort Study

**DOI:** 10.3390/jcm15124617

**Published:** 2026-06-14

**Authors:** Dinçer Sümer, Gunel Aliyeva, Ümran Özcan, Bengisu Elüstü, İslam Aslanlı, Beyza Nur Aslan, Belgin Savran Üçok, Zehra Vural Yılmaz

**Affiliations:** 1 Department of Perinatology, University of Health Sciences, Etlik City Hospital, Varlık Mahallesi Halil Sezai Erkut Cd. No:5, 06170 Ankara, Türkiye; dr.aliyeva87@gmail.com (G.A.); ozcanumran38@gmail.com (Ü.Ö.); bengisucakr@gmail.com (B.E.); zehravural@gmail.com (Z.V.Y.); 2 Department of Obstetrics and Gynecology, University of Health Sciences, Etlik City Hospital, Varlık Mahallesi Halil Sezai Erkut Cd. No:5, 06170 Ankara, Türkiye; islamaslanli80@gmail.com (İ.A.); drbeyzanuraslan@gmail.com (B.N.A.); dr.belgin@gmail.com (B.S.Ü.)

**Keywords:** flat OGTT, oral glucose tolerance test, small-for-gestational-age, fetal growth restriction

## Abstract

**Objective:** To evaluate the clinical significance of a flat oral glucose tolerance test (OGTT) pattern and its association with obstetric and perinatal outcomes, focusing on fetal growth. **Methods:** This retrospective cohort study was conducted at a tertiary referral center between January 2023 and November 2025. A total of 1198 women who underwent a two-step OGTT between 24 and 32 weeks of gestation were screened, and 685 eligible participants were included. A flat OGTT pattern was defined as fasting glucose < 95 mg/dL with all postprandial values < 100 mg/dL. The primary outcome was small-for-gestational-age (SGA); secondary outcomes included fetal growth restriction (FGR) and other obstetric outcomes. Logistic regression analyses were performed to assess independent associations. **Results:** Thirty-nine women (5.7%) exhibited a flat OGTT pattern. These pregnancies were characterized by a markedly attenuated postprandial glycemic response and lower fasting glucose levels. Most maternal and neonatal outcomes were similar between groups. However, SGA was significantly more frequent in the flat OGTT group (20.5% vs. 6.7%, *p* = 0.001). In multivariable analysis, a flat OGTT pattern remained independently associated with SGA (aOR 4.05, 95% CI 1.70–9.68, *p* = 0.002). Both SGA and FGR were more frequent among women with a flat OGTT pattern, although the association appeared stronger for SGA. **Conclusions:** A flat OGTT pattern appears to represent a distinct glycemic response characterized by an attenuated postprandial glucose response and lower fasting glucose levels. Although this pattern was not associated with a generalized increase in adverse obstetric or neonatal outcomes, it was associated with an increased risk of small-for-gestational-age infants.

## 1. Introduction

Gestational diabetes mellitus (GDM) is defined as glucose intolerance with onset or first recognition during pregnancy and affects approximately 2–38% of pregnancies worldwide [1,2]. GDM is associated with an increased risk of adverse maternal and neonatal outcomes, including hypertensive disorders of pregnancy, fetal macrosomia, operative delivery, and neonatal respiratory complications [3,4]. Therefore, screening for GDM is routinely performed between 24 and 28 weeks of gestation using either a one-step or two-step oral glucose tolerance test (OGTT) approach [5,6]. In the two-step approach, a 50 g glucose challenge test is performed first, followed by a diagnostic 100 g OGTT if the 1 h plasma glucose level is ≥140 mg/dL. Diagnostic thresholds for the 100 g OGTT are fasting ≥ 95 mg/dL, 1 h ≥ 180 mg/dL, 2 h ≥ 155 mg/dL, and 3 h ≥ 140 mg/dL, with at least two abnormal values required for the diagnosis of GDM [7].

A typical OGTT response features a low fasting glucose level, followed by a rise in plasma glucose concentrations that peaks within 30 to 60 min after glucose ingestion and then gradually declines [8]. However, not all individuals exhibit this expected glycemic pattern. A flat OGTT curve, defined by a blunted or absent postprandial glucose rise, represents a distinct and less well-understood glycemic response. There is currently no universally accepted definition of a flat OGTT pattern. It has been described as either a less than 16.5% increase in plasma glucose levels during the test or as a pattern with fasting glucose < 95 mg/dL and all postprandial values remaining below 100 mg/dL [9,10].

The clinical implications of a flat OGTT pattern during pregnancy remain controversial. Some studies have suggested an association with lower birth weight, increased rates of small-for-gestational-age (SGA) infants, and lower Apgar scores [11], while others have reported a more favorable metabolic profile, including reduced risks of large-for-gestational-age infants, macrosomia, and hypertensive disorders of pregnancy [9]. These inconsistent findings may be due to differences in study design, population characteristics, and the lack of a standardized definition of a flat OGTT pattern.

The pathophysiological basis of a flat OGTT pattern remains incompletely understood. In nonpregnant individuals, a flat OGTT curve is generally considered a variant of normal carbohydrate tolerance, although it has also been linked to endocrine and metabolic abnormalities. Proposed mechanisms include an exaggerated insulin response resulting in rapid glucose disposal and an attenuated postprandial glycemic excursion [12]. From a physiological perspective, maternal glucose is the primary energy substrate for the fetus and is transferred across the placenta through facilitated diffusion [13,14]. Fetal glucose concentrations largely depend on maternal glucose availability, and alterations in maternal glycemic patterns may influence fetal nutrient supply and growth [15,16]. While postprandial hyperglycemia has been extensively linked to fetal overgrowth and macrosomia, the potential implications of an attenuated maternal glycemic response remain less well understood [15,16]. A blunted postprandial glucose excursion could theoretically reduce transplacental glucose availability and thereby influence fetal growth trajectories. Consistent with this hypothesis, previous studies have reported lower birth weight and increased rates of small-for-gestational-age infants among pregnancies exhibiting a flat OGTT pattern [11]. This biological rationale provides a plausible basis for investigating whether a flat OGTT pattern is associated with impaired fetal growth outcomes, including small-for-gestational-age infants.

Most previous studies have focused on broad obstetric outcomes, and the potential selective impact of a flat OGTT pattern, particularly on fetal growth, has not been clearly defined. Additionally, data on its association with fetal growth outcomes after adjustment for maternal characteristics remain limited. Therefore, this study aimed to evaluate the clinical characteristics of pregnancies with a flat OGTT pattern and to investigate its association with obstetric and perinatal outcomes, with a particular focus on fetal growth parameters.

## 2. Materials and Methods

### 2.1. Study Design and Patient Selection

This retrospective cohort study was conducted at the Perinatology Clinic of Etlik City Hospital, Ankara, Türkiye, a tertiary referral center, between January 2023 and November 2025. During the study period, 38,283 deliveries occurred at the institution.

A total of 1198 pregnant women who underwent a two-step oral glucose tolerance test between 24 and 32 weeks of gestation were initially screened. Clinical, laboratory, ultrasonographic, and perinatal outcome data were retrospectively extracted from patients’ medical records and the hospital’s electronic medical database by the investigators.

The two-step screening approach consisted of a 50 g glucose challenge test, followed by a diagnostic 100 g OGTT in cases with a 1 h plasma glucose level ≥ 140 mg/dL. All OGTTs were performed after an overnight fast of at least 8 h, with blood samples collected at fasting and at 1, 2, and 3 h after glucose ingestion, in accordance with standard clinical protocols.

Participants were classified based on OGTT results according to the Carpenter and Coustan (CC) criteria [7]. A flat OGTT pattern was defined as a fasting plasma glucose level < 95 mg/dL with all postprandial glucose values remaining below 100 mg/dL, consistent with definitions used in previous studies.

Exclusion criteria were applied to ensure a homogeneous study population and minimize potential confounding factors affecting glucose metabolism and pregnancy outcomes. These included multiple pregnancy, loss to follow-up, OGTT performed before 24 weeks of gestation, delivery outside the study institution, pre-existing diabetes mellitus or chronic hypertension, inflammatory or autoimmune diseases, malignancy, corticosteroid usage, fetal chromosomal or structural anomalies, incomplete OGTT data, and a diagnosis of gestational diabetes mellitus.

**Figure 1:**
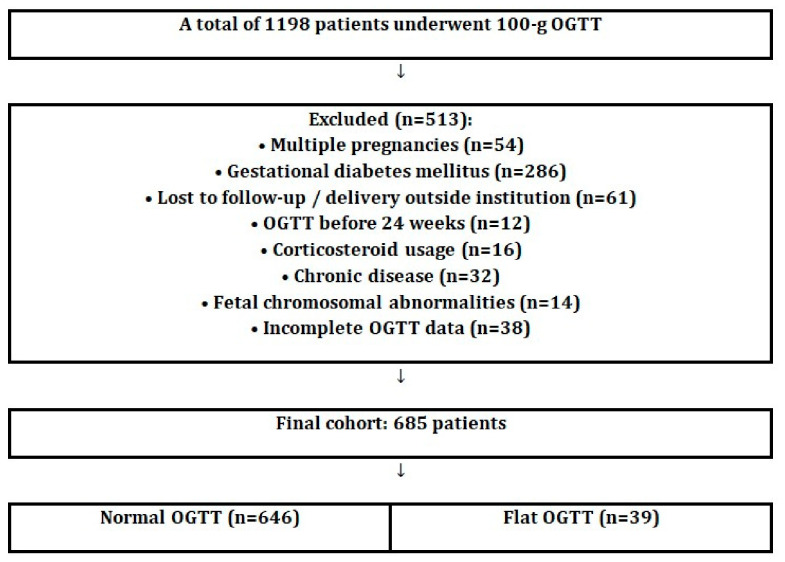
Flow diagram of the study population. A total of 1198 women who underwent a two-step oral glucose tolerance test (OGTT) were screened. After applying exclusion criteria, 685 women were included in the final analysis, comprising 646 with normal OGTT and 39 with a flat OGTT pattern.

After applying the predefined criteria, 685 women were included in the final analysis: 39 with a flat OGTT pattern and 646 with normal OGTT results. A flow diagram of the study population is presented in Figure 1.

Ethical approval was obtained from the Ankara Etlik City Hospital Ethics Committee (AEŞH-BADEK2-2025-724, Date: 16 December 2025), and the study was conducted in accordance with the principles of the Declaration of Helsinki. Given the retrospective design, the requirement for informed consent was waived by the ethics committee.

No prospective sample size calculation was performed due to the retrospective design, and the sample size was determined by the number of eligible patients available during the study period.

This study was conducted and reported in accordance with the Strengthening the Reporting of Observational Studies in Epidemiology (STROBE) statement for observational studies.

### 2.2. Definition of Terms

Ultrasonographic examinations were conducted using a Voluson S10 system (General Electric, Boston, MA, USA) to determine gestational age and assess fetal viability. Gestational age was primarily confirmed by crown–rump length measurement obtained during the first-trimester ultrasound examination. Fetal biometric parameters were assessed according to the guidelines of the International Society of Ultrasound in Obstetrics and Gynecology (ISUOG) [17,18,19,20]. All ultrasonographic examinations were performed by experienced obstetricians following a standardized protocol.

Oligohydramnios was defined as a maximum vertical pocket of less than 2 cm and polyhydramnios as a maximum vertical pocket of 8 cm or greater [21]. Preterm birth was defined as delivery before 37 weeks of gestation [22] and macrosomia as a birth weight greater than 4000 g. Fetal growth restriction (FGR) was defined according to Delphi/ISUOG criteria, while small-for-gestational-age (SGA) was defined as birth weight below the 10th percentile for gestational age, based on the Hadlock fetal growth reference charts, in accordance with current international recommendations [19,20,23,24,25].

Hypertensive disorders of pregnancy, including gestational hypertension and preeclampsia, were defined according to the American College of Obstetricians and Gynecologists (ACOG) guidelines [26]. Intrahepatic cholestasis of pregnancy was defined as new-onset pruritus with elevated serum bile acid levels [27]. Preterm premature rupture of membranes was defined as rupture of the fetal membranes before labor and before 37 weeks of gestation [28]. Placental abruption was defined as partial or complete separation of the placenta from the decidua after 20 weeks of gestation [29].

The primary outcome of the study was the occurrence of small-for-gestational-age infants. Secondary outcomes included fetal growth restriction, hypertensive disorders of pregnancy, preterm birth, and other adverse obstetric and neonatal outcomes.

### 2.3. Statistical Analysis

Statistical analyses were conducted using the Statistical Package for the Social Sciences (SPSS) version 22.0 (IBM Corp., Armonk, NY, USA). A two-sided *p*-value < 0.05 was considered statistically significant.

The distribution of continuous variables was assessed using the Shapiro–Wilk and Kolmogorov–Smirnov tests, as appropriate. Continuous variables were reported as mean ± standard deviation (SD) or median with interquartile range (IQR), depending on distribution. Between-group comparisons were performed using the independent samples *t*-test or Mann–Whitney U test for continuous variables, and the chi-square test or Fisher’s exact test for categorical variables.

To evaluate the association between a flat OGTT pattern and adverse fetal growth outcomes, univariate and multivariable logistic regression analyses were conducted for small-for-gestational-age infants as the primary outcome and fetal growth restriction as a secondary outcome. Variables included in the multivariable models were selected based on clinical relevance and prior literature, including maternal age, first-trimester body mass index (BMI), gestational weight gain, and gravidity. The strength of associations was expressed as odds ratios (ORs) and adjusted odds ratios (aORs) with corresponding 95% confidence intervals (CIs).

To improve model interpretability and avoid redundancy, parity was excluded from the multivariable analysis because of its conceptual overlap with gravidity. Collinearity diagnostics were performed using variance inflation factors (VIFs). First-trimester BMI and BMI at delivery demonstrated moderate collinearity; therefore, only first-trimester BMI was retained in the multivariable models because it was considered more clinically relevant to the study objective.

## 3. Results

Baseline maternal demographic and clinical characteristics were comparable between the flat OGTT and normal OGTT groups. There were no statistically significant differences in maternal age, gravidity, parity, first-trimester body mass index (BMI), BMI at delivery, gestational weight gain, or gestational age at the time of OGTT (all *p* > 0.05).

Maternal age tended to be lower in the flat OGTT group; however, this difference was not statistically significant (median 26 vs. 29 years, *p* = 0.069). Similarly, BMI at delivery showed a borderline difference, with slightly lower values in the flat OGTT group, but this was not statistically significant (*p* = 0.053).

As expected based on the predefined criteria for a flat OGTT pattern, both fasting and postprandial glucose levels were significantly lower in the flat OGTT group compared to the normal OGTT group. Fasting glucose levels were modestly lower (median 73 vs. 78 mg/dL, *p* = 0.007), while the most pronounced differences were observed in postprandial measurements. The flat OGTT group demonstrated consistently attenuated glucose responses at all post-load time points, with markedly lower 1 h, 2 h, and 3 h glucose levels (all *p* < 0.001) (Table 1).

**Figure 2:**
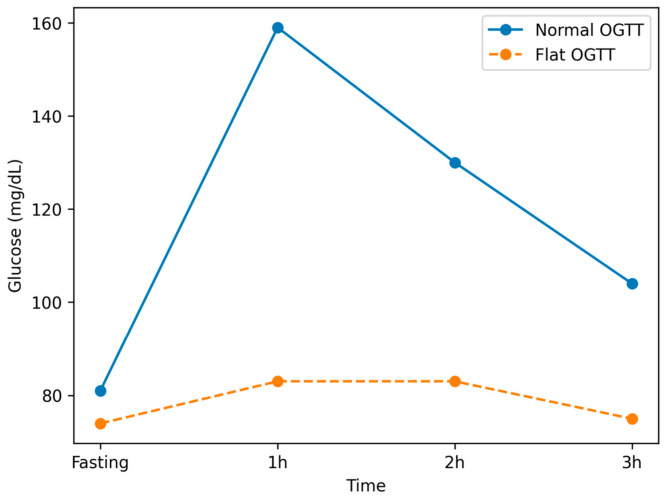
Mean glucose levels during oral glucose tolerance testing in the normal and flat OGTT groups. The normal OGTT group demonstrated a typical postprandial glucose peak, whereas the flat OGTT group exhibited a blunted glycemic response across all time points. Values are presented as mean ± standard error of the mean (SEM).

The graphical representation of OGTT responses clearly shows a typical postprandial glucose peak in the normal OGTT group, whereas the flat OGTT group exhibited a markedly blunted glycemic response across all time points (Figure 2).

Obstetric outcomes are presented in Table 2. Overall, most obstetric outcomes were comparable between the flat OGTT and normal OGTT groups. There were no statistically significant differences in the rates of polyhydramnios, oligohydramnios, preterm labor, preterm premature rupture of membranes, placental abruption, intrauterine fetal demise, gestational hypertension, preeclampsia, or macrosomia (all *p* > 0.05).

However, fetal growth–related outcomes differed between the groups. The incidence of fetal growth restriction was significantly higher in the flat OGTT group compared with the normal OGTT group (10.3% vs. 3.1%, *p* = 0.042). More notably, the rate of small-for-gestational-age (SGA) infants was markedly increased in the flat OGTT group (20.5% vs. 6.7%, *p* = 0.001).

Although intrahepatic cholestasis of pregnancy appeared to be more frequent in the flat OGTT group (5.1% vs. 1.1%), this difference did not reach statistical significance (*p* = 0.088). Similarly, intrauterine fetal demise was numerically higher in the flat OGTT group; however, this finding was not statistically significant.

In univariate logistic regression analysis, gestational weight gain was significantly associated with FGR, showing a protective effect (OR: 0.895, 95% CI: 0.815–0.984, *p* = 0.021). Maternal age, gravidity, fetal sex, and first-trimester BMI were not significantly associated with FGR (all *p* > 0.05). A flat OGTT pattern was significantly associated with increased risk of FGR in univariate analysis (OR: 3.577, 95% CI: 1.160–11.032, *p* = 0.027). In multivariable analysis adjusting for maternal age, gravidity, fetal sex, first-trimester BMI, and gestational weight gain, the association between a flat OGTT pattern and FGR remained statistically significant (aOR: 4.313, 95% CI: 1.312–14.171, *p* = 0.016). Gestational weight gain showed a borderline association with reduced risk (aOR: 0.908, 95% CI: 0.822–1.003, *p* = 0.056), while maternal age, gravidity, fetal sex, and first-trimester BMI were not independently associated with FGR (Table 3).

In univariate logistic regression analysis, gestational weight gain was inversely associated with the risk of small-for-gestational-age infants (OR: 0.941, 95% CI: 0.886–0.999, *p* = 0.048), suggesting a potential protective effect. Maternal age, gravidity, fetal sex, and first-trimester BMI were not significantly associated with SGA (all *p* > 0.05). A flat OGTT pattern was strongly associated with an increased risk of SGA in univariate analysis (OR: 3.613, 95% CI: 1.565–8.340, *p* = 0.003).

In multivariable logistic regression analysis adjusting for maternal age, gravidity, fetal sex, first-trimester BMI, and gestational weight gain, the flat OGTT pattern remained independently associated with SGA (aOR: 4.059, 95% CI: 1.701–9.685, *p* = 0.002). Gestational weight gain was no longer statistically significant after adjustment, although a trend toward a protective effect persisted (aOR: 0.951, 95% CI: 0.892–1.013, *p* = 0.116). Maternal age, gravidity, fetal sex, and first-trimester BMI were not independently associated with SGA (Table 4).

Neonatal outcomes are shown in Table 5. Neonatal and delivery outcomes were largely similar between the flat OGTT and normal OGTT groups. There were no statistically significant differences in gestational age at delivery (median 39 vs. 39 weeks, *p* = 0.734) or birth weight (median 3180 vs. 3260 g, *p* = 0.082).

The mode of delivery was also similar between groups, with comparable rates of cesarean section and vaginal birth (*p* = 0.321). Neonatal characteristics, including sex distribution, 1 min and 5 min Apgar scores, and NICU admission rates, were also similar between the groups (all *p* > 0.05).

## 4. Discussion

In this retrospective cohort study, we evaluated the clinical significance of a flat OGTT pattern and its association with obstetric and perinatal outcomes. The main findings are as follows. First, pregnancies with a flat OGTT pattern showed a markedly attenuated postprandial glycemic response and significantly lower fasting glucose levels compared to the normal OGTT group. Second, this distinct glycemic response pattern was not associated with an increased risk of most adverse maternal or obstetric outcomes. Third, the rate of small-for-gestational-age (SGA) infants was significantly higher in the flat OGTT group, and this association remained robust after adjustment for maternal characteristics.

Both SGA and fetal growth restriction (FGR) were more frequent among women with a flat OGTT pattern, although the association was stronger for SGA. Overall, these findings suggest that a flat OGTT pattern may be associated with impaired fetal growth rather than a generalized increase in adverse pregnancy outcomes.

Previous studies evaluating the clinical implications of a flat OGTT pattern have reported varied findings. In a large cohort of 14,122 pregnant women, 6.8% were identified as having a flat OGTT pattern; these women were younger, had lower body mass index, and showed lower rates of hypertension. After adjustment for maternal age, obesity, and hypertension, a flat OGTT pattern was associated with a reduced long-term risk of type 2 diabetes mellitus up to five years postpartum [10]. Similarly, a retrospective cohort of 2673 women found that a flat OGTT curve was associated with lower mean birth weight, higher rates of small-for-gestational-age infants, and lower 5 min Apgar scores, without significant differences in overall obstetric or maternal outcomes [30]. Additional large-scale studies have suggested a more favorable metabolic and obstetric profile, including lower risks of macrosomia, large-for-gestational-age infants, and hypertensive disorders of pregnancy [9,31]. In contrast, isolated reports have suggested a possible association with adverse outcomes such as stillbirth; however, these findings are limited by small sample sizes and should be interpreted with caution [32].

Consistent with studies reporting lower birth weight and increased SGA rates, our findings showed a significantly higher rate of SGA in the flat OGTT group. Most maternal and neonatal outcomes, including hypertensive disorders of pregnancy and NICU admission, were similar between groups. The flat OGTT group also had lower fasting glucose levels, supporting the presence of a distinct glycemic phenotype rather than a simple variation within normal glucose responses. Both SGA and fetal growth restriction (FGR) were more frequent among women with a flat OGTT pattern, and both associations remained significant after multivariable adjustment. However, the magnitude of the association appeared stronger for SGA, suggesting that the impact of a flat OGTT pattern may be more pronounced for SGA than for FGR.

The underlying mechanisms of a flat OGTT pattern during pregnancy are not fully understood. One possible explanation is that this pattern indicates increased maternal insulin sensitivity or more efficient peripheral glucose uptake, leading to reduced postprandial glycemic excursions. In this context, postprandial glycemia—rather than fasting glucose alone—has been identified as a key determinant of adverse obstetric outcomes, particularly fetal overgrowth and metabolic complications [15,16]. Alternatively, a flat OGTT pattern may indicate reduced glycemic variability, which has been linked to more favorable metabolic profiles and lower oxidative stress [33]. Reduced glucose fluctuations may help prevent endothelial dysfunction, a central mechanism in hypertensive disorders of pregnancy [34]. Conversely, a blunted glycemic response could reflect subtle changes in maternal–placental nutrient transfer, potentially limiting fetal substrate availability [11]. In our cohort, the combination of generally preserved obstetric outcomes with an increased rate of SGA supports the hypothesis of a selective effect on fetal growth rather than a generalized adverse profile.

From a clinical perspective, our findings indicate that a flat OGTT pattern is not linked to a general increase in adverse obstetric outcomes. However, the consistent association with SGA suggests that this phenotype may require closer monitoring of fetal growth. In routine clinical practice, atypical OGTT patterns can create uncertainty; our results indicate that a flat glycemic response alone should not lead to unnecessary interventions but may support a more individualized, risk-based approach, including consideration of serial fetal growth assessments. The consistency, magnitude, and independence of the association suggest that a flat OGTT pattern may serve as a potential marker that requires further validation for identifying pregnancies at increased risk of SGA.

This study has several limitations. First, its retrospective design introduces inherent issues, including selection bias and residual confounding. Second, the relatively small number of patients with a flat OGTT pattern may have limited the ability to detect associations with rare outcomes. No prospective sample size calculation was performed due to the retrospective design; the study size was determined by the number of eligible patients available during the study period. Additionally, the small number of FGR events in the flat OGTT group may have affected the precision and stability of the regression estimates. Third, the relatively wide confidence intervals for some estimates indicate limited precision, likely related to the imbalance in group sizes. Fourth, because multiple maternal and neonatal outcomes were evaluated, the possibility of type I error due to multiple comparisons cannot be excluded, so the findings should be interpreted with appropriate caution. Finally, long-term maternal and offspring metabolic outcomes were not available, preventing evaluation of the long-term clinical implications of a flat OGTT pattern.

Despite these limitations, this study has several notable strengths. To our knowledge, it is among the few studies to evaluate the clinical implications of a flat OGTT pattern using a clearly defined and clinically applicable diagnostic criterion. The use of a well-characterized cohort from a large tertiary referral center enhances the clinical relevance and generalizability of the findings. In addition, the comprehensive assessment of both obstetric and neonatal outcomes provides a broad evaluation of potential clinical implications. The exclusion of women with GDM allowed for a more homogeneous comparison, reducing confounding related to overt dysglycemia. Finally, the use of multivariable analysis to adjust for key maternal characteristics strengthens the validity of the observed independent association between a flat OGTT pattern and SGA.

## 5. Conclusions

In conclusion, a flat OGTT pattern may represent a distinct glycemic response characterized by an attenuated postprandial glucose excursion and lower fasting glucose levels. Although this pattern was not associated with a general increase in adverse obstetric or neonatal outcomes, it was linked to a higher risk of SGA and FGR in this cohort. These findings suggest that a flat OGTT pattern may be associated with impaired fetal growth rather than indicating a uniformly benign or pathological condition. However, the observational nature of the study precludes conclusions about causality, underlying mechanisms, or clinical utility. Further large-scale prospective studies are needed to validate these findings, clarify the biological mechanisms involved, and determine their potential clinical relevance.

## Figures and Tables

**Table 1:** Maternal demographic, clinical, and glycemic characteristics of the study groups.

Parameter	Normal OGTT (n = 646)	Flat OGTT (n = 39)	*p* Value
Age (years)	29.0 (8.0)	26.0 (5.0)	0.069
Gravidity	2.0 (2.0)	2.0 (1.0)	0.236
Parity	1.0 (1.0)	1.0 (1.0)	0.200
BMI at first trimester (kg/m^2^)	26.0 (7.0)	24.0 (7.0)	0.140
BMI at delivery (kg/m^2^)	30.0 (7.0)	28.0 (9.0)	0.053
Weight gain during pregnancy (kg)	10.0 (7.0)	10.0 (9.0)	0.675
Gestational age at OGTT (weeks)	26.0 (1.0)	26.0 (2.0)	0.333
Fasting glucose level during OGTT (mg/dL)	78.0 (10.0)	73.0 (12.0)	*0.007*
One-hour plasma glucose level (mg/dL)	147.0 (36.0)	87.0 (22.0)	*<0.001*
Two-hour plasma glucose level (mg/dL)	117.0 ± 24.4	77.4 ± 13.4	*<0.001*
Three-hour plasma glucose level (mg/dL)	97.0 (41.0)	67.5 (23.0)	*<0.001*

Continuous variables are presented as mean ± standard deviation (SD) or median (interquartile range, IQR), depending on data distribution. Statistically significant values are presented in italics. BMI: body mass index; OGTT: oral glucose tolerance test.

**Table 2:** Comparison of obstetric and fetal growth outcomes between the normal and flat OGTT groups.

Parameter	Normal OGTT (n = 646)	Flat OGTT (n = 39)	*p* Value
Polyhydramnios (n, %)	26 (4.0%)	2 (5.1%)	0.670
Oligohydramnios (n, %)	41 (6.3%)	1 (2.6%)	0.503
Fetal growth restriction (FGR) (n, %)	20 (3.1%)	4 (10.3%)	*0.042*
Small-for-Gestational-Age (SGA) (n, %)	43 (6.7%)	8 (20.5%)	*0.001*
Preterm labor (n,%)	16 (2.5%)	1 (2.6%)	1.000
Preterm Premature Rupture of Membranes (PPROM) (n, %)	13 (2.0%)	0 (0.0%)	1.000
Placental abruption (n, %)	1 (0.2%)	0 (0.0%)	1.000
Intrauterine fetal demise (n, %)	3 (0.5%)	1 (2.6%)	0.209
Gestational hypertension (n, %)	22 (3.4%)	1 (2.6%)	1.000
Preeclampsia (n, %)	9 (1.4%)	0 (0.0%)	1.000
Intrahepatic cholestasis of pregnancy (n, %)	7 (1.1%)	2 (5.1%)	0.088
Macrosomia (>4000 g, n, %)	35 (5.4%)	0 (0.0%)	0.251

Categorical variables are presented as number (percentage). Statistically significant values are presented in italics. FGR: fetal growth restriction; SGA: small-for-gestational-age; PPROM: preterm premature rupture of membranes; OGTT: oral glucose tolerance test.

**Table 3:** Univariate and multivariable logistic regression analysis of factors associated with fetal growth restriction (FGR).

		Univariate			Multivariate	
Parameter	OR	95% CI	*p* Value	aOR	95% CI	*p* Value
Age	0.984	0.912–1.062	0.684	0.976	0.890–1.069	0.597
Gravidity	1.128	0.866–1.470	0.371	1.216	0.881–1.677	0.234
Fetal sex	0.946	0.418–2.143	0.895	0.851	0.355–2.041	0.717
BMI at first trimester	0.964	0.886–1.050	0.400	0.972	0.889–1.062	0.525
Weight gain during pregnancy	0.895	0.815–0.984	*0.021*	0.908	0.822–1.003	0.056
Flat OGTT	3.577	1.160–11.032	*0.027*	4.313	1.312–14.171	*0.016*

OR: odds ratio; aOR: adjusted odds ratio; CI: confidence interval; BMI: body mass index; OGTT: oral glucose tolerance test. Statistically significant values are presented in italics.

**Table 4:** Univariate and multivariable logistic regression analysis of factors associated with small-for-gestational-age (SGA).

		Univariate			Multivariate	
Parameter	OR	95% CI	*p* Value	aOR	95% CI	*p* Value
Age	0.987	0.935–1.041	0.621	0.987	0.927–1.050	0.680
Gravidity	1.011	0.826–1.237	0.917	1.074	0.843–1.369	0.563
Fetal sex	1.520	0.855–2.703	0.154	1.491	0.814–2.731	0.196
BMI at first trimester	0.991	0.936–1.049	0.747	0.988	0.940–1.059	0.941
Weight gain during pregnancy	0.941	0.886–0.999	*0.048*	0.951	0.892–1.013	0.116
Flat OGTT	3.613	1.565–8.340	*0.003*	4.059	1.701–9.685	*0.002*

OR: odds ratio; aOR: adjusted odds ratio; CI: confidence interval; BMI: body mass index; OGTT: oral glucose tolerance test. Statistically significant values are presented in italics.

**Table 5:** Comparison of neonatal and delivery outcomes between normal and flat OGTT groups.

Parameter	Normal OGTT (n = 646)	Flat OGTT (n = 39)	*p* Value
Gestational age at delivery (weeks)	39 (2)	39 (2)	0.734
Birth weight (g)	3260 (560)	3180 (740)	0.082
Mode of delivery Cesarean section (n, %) Vaginal birth (n, %)	370 (57.3%) 276 (42.7%)	20 (51.3%) 19 (48.7%)	0.321
Fetal sex Female Male	301 (46.6%) 345 (53.4%)	22 (56.4%) 17 (43.6%)	0.251
1 min Apgar score	9 (1)	9 (1)	0.819
5 min Apgar score	10 (1)	10 (1)	0.478
NICU admission (n, %)	21 (3.3%)	1 (2.6%)	1.000

Continuous variables are presented as median (interquartile range, IQR). Categorical variables are presented as number (percentage). NICU: neonatal intensive care unit; OGTT: oral glucose tolerance test.

## Data Availability

The datasets used and/or analyzed during the current study are available from the corresponding author on reasonable request.

## List of Abbreviations

aORs	Adjusted odds ratios
ACOG	American College of Obstetricians and Gynecologists
BMI	Body Mass Index
CC	Carpenter and Coustan
FGR	Fetal Growth Restriction
GDM	Gestational diabetes mellitus
ISUOG	International Society of Ultrasound in Obstetrics and Gynecology
IQR	Interquartile ranges
NICU	Neonatal Intensive Care Unit
ORs	Odds ratios
OGTT	Oral glucose tolerance test
SGA	Small-for-gestational-age
SD	Standard deviation
SEM	Standard error of the mean
STROBE	Strengthening the Reporting of Observational Studies in Epidemiology
VIF	Variance inflation factors
CIs	95% confidence intervals
